# Supplementary material for: Comparative Analysis of Original and Replaced Gut Microbiomes within Same Individuals Identified the Intestinal Microbes Associated with Weight Gaining
Source: Microorganisms. 2022 May 20;10(5):1062. doi: 10.3390/microorganisms10051062 (PMC9144321; doi:10.3390/microorganisms10051062)
Supplement: Supplementary file 1 [file microorganisms-10-01062-s001.zip › microorganisms-1648546-supplementary.pdf]

Supplementary Materials

Supplemental Figures

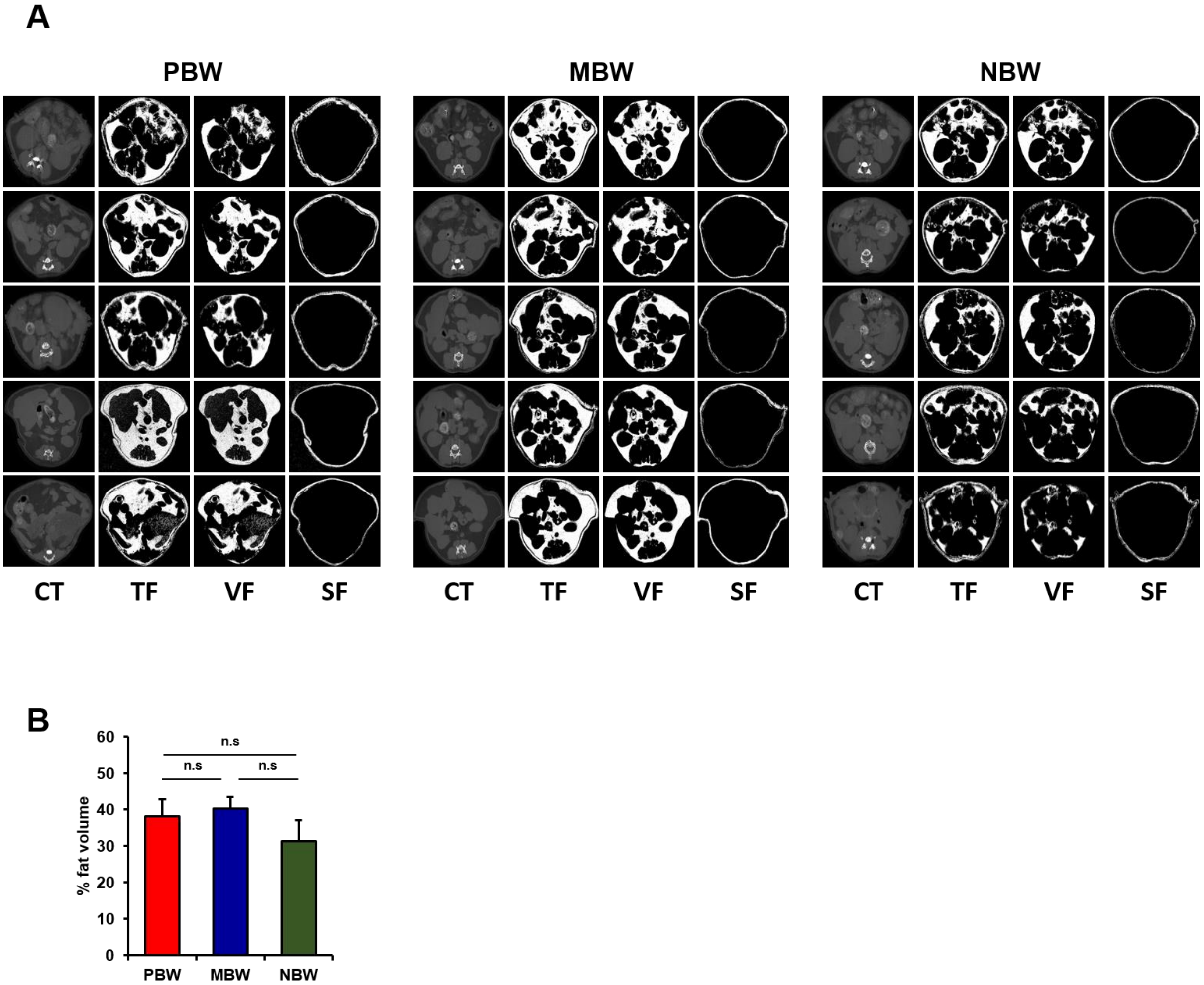

Figure S1. The representative micro-CT image of each experimental mouse of the PBW, MBW, and NBW groups three months after the gut microbiome replacement. (A) Micro-CT image, **(B)** The fat volume change of each group. TF, total fat; VF, visceral fat; SF, subcutaneous fat. The values are represented as mean ± SEM. *n.s:* not significant ($p > 0.05$).

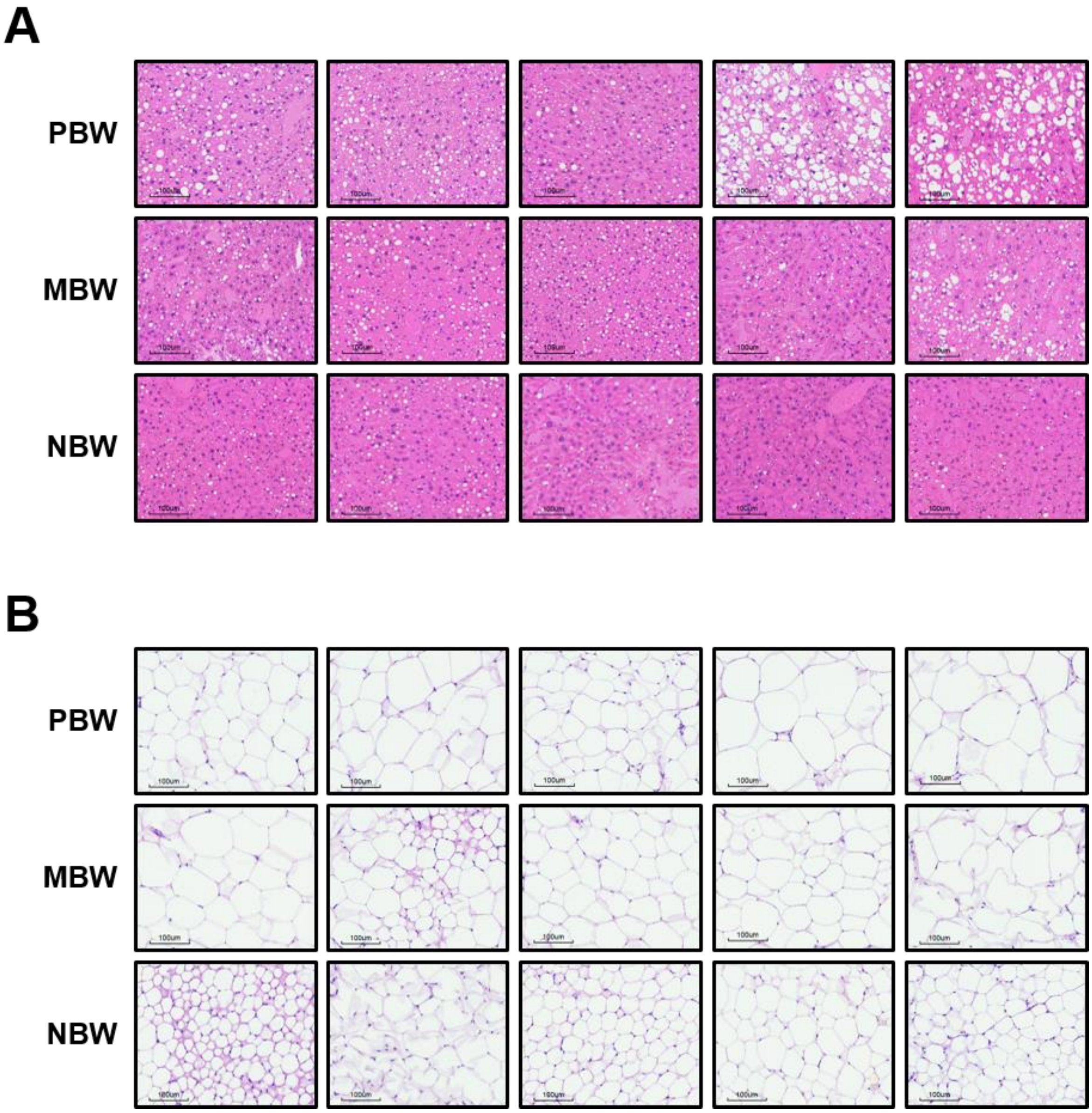

Figure S2. The representative histological images of each experimental mouse of the PBW, MBW, and NBW groups three months after gut microbiome replacement. (A) The H&E-stained liver images. **(B)** The H&E-stained adipose tissue images. Scale bars, 100 μm in all the images.

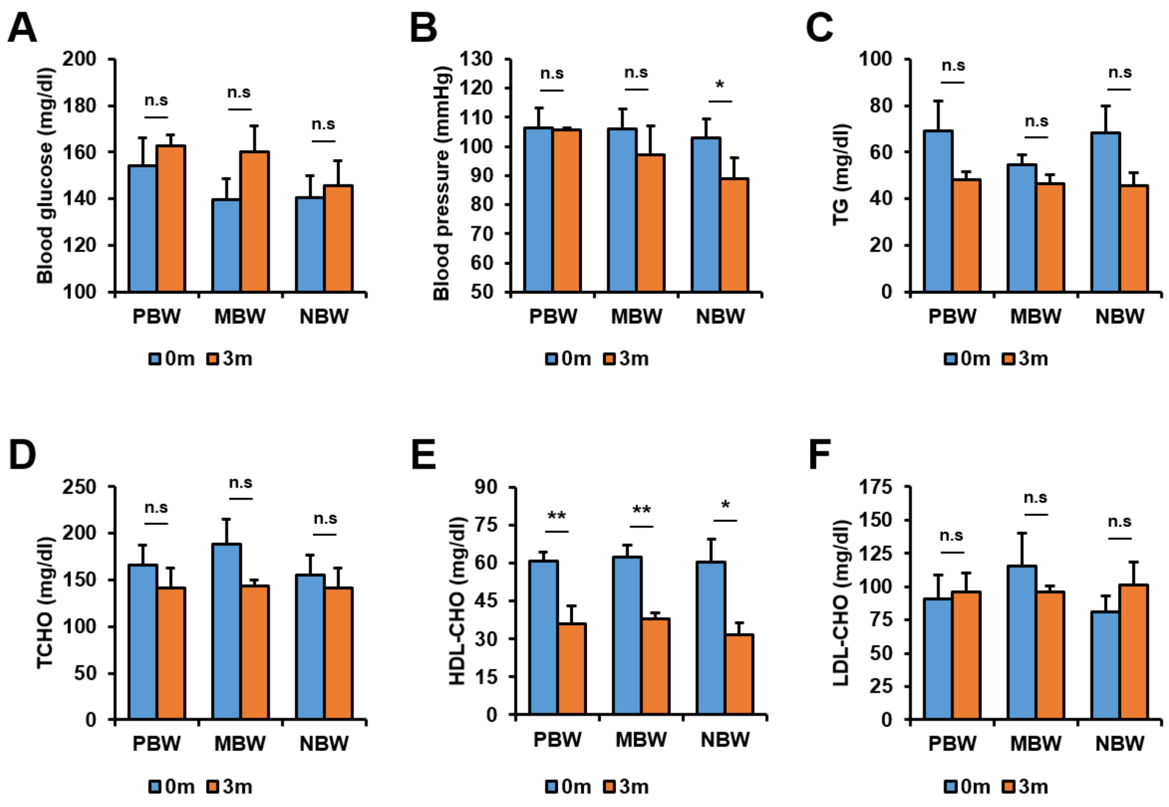

Figure S3. The effects of the gut microbiome replacement on the changes in the blood glucose, blood pressure, and lipid levels. The average values of **(A)** Blood glucose, **(B)** Blood pressure, **(C)** Total glycerol (TG), **(D)** Total cholesterol (TCHO), **(E)** HDL-cholesterol (HDL-CHO) **(F)** LDL-cholesterol (LDL-CHO) of each group are shown. The values are represented as mean ± SEM. 0m represents before the gut microbiome replacement, and 3m represents 3 months after the gut microbiome replacement. *$p < 0.05$; **$p < 0.01$; *n.s:* not significant ($p > 0.05$).

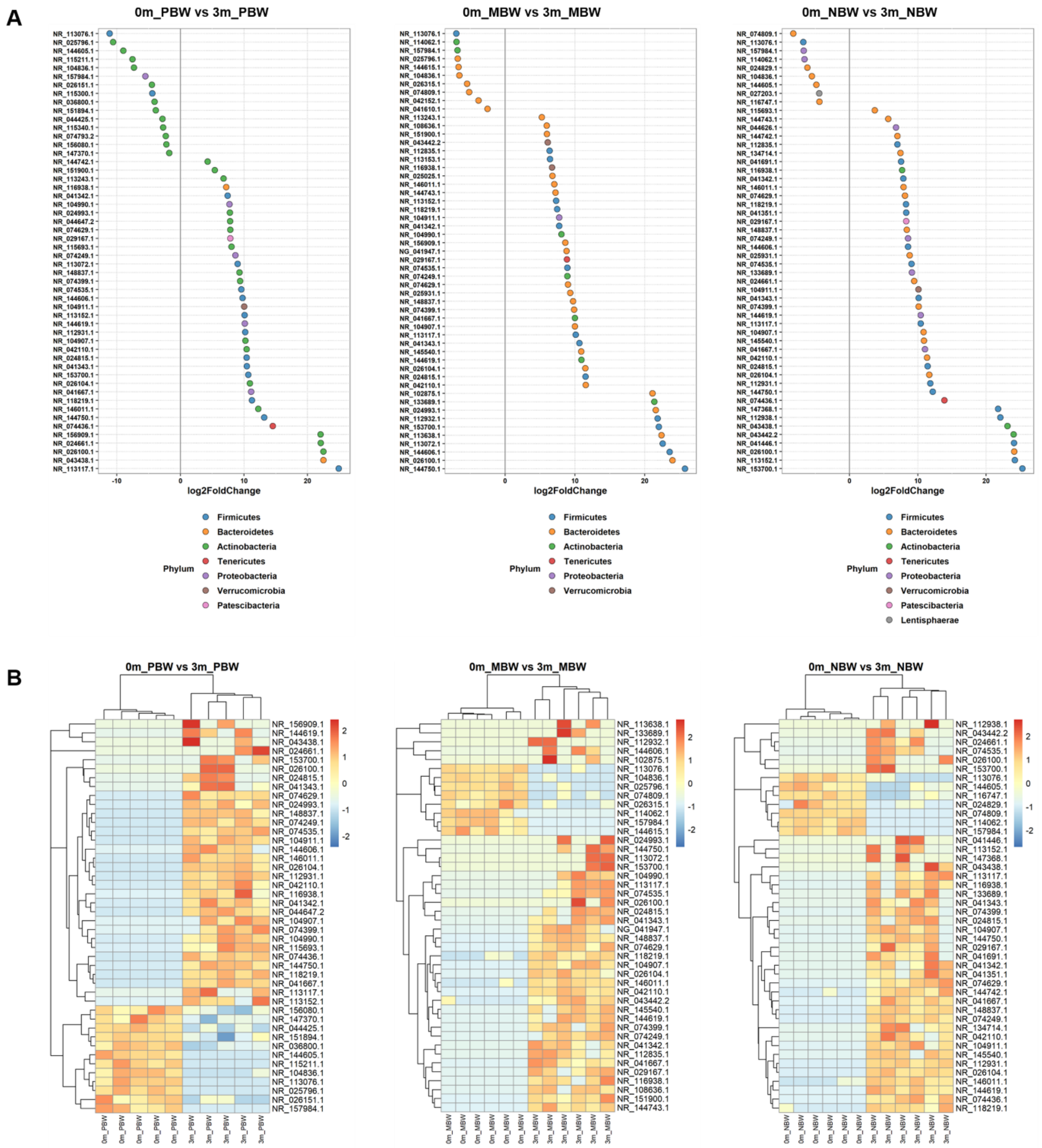

A
0m_PBW vs 3m_PBW
0m_MBW vs 3m_MBW
0m_NBW vs 3m_NBW
log2FoldChange
Phylum
Firmicutes
Bacteroidetes
Actinobacteria
Tenericutes
Proteobacteria
Verrucomicrobia
Patescibacteria
Lentisphaerae
B
0m_PBW vs 3m_PBW
0m_MBW vs 3m_MBW
0m_NBW vs 3m_NBW

Figure S4. Differential abundance analysis of species changes of each group before and after the gut microbiome replacement. (A) Dotplots showing significantly differentially abundant OTUs, where OTUs are sorted by species along the y-axis and colored by phylum, analyzed by DESeq2 differential abundance analysis. The x-axis indicates the log2 fold-change in 3 month of each group compared to 0 month of each groups as baseline. **(B)** Heatmaps of significantly differentially abundant species in 0 month and 3 month of each group.

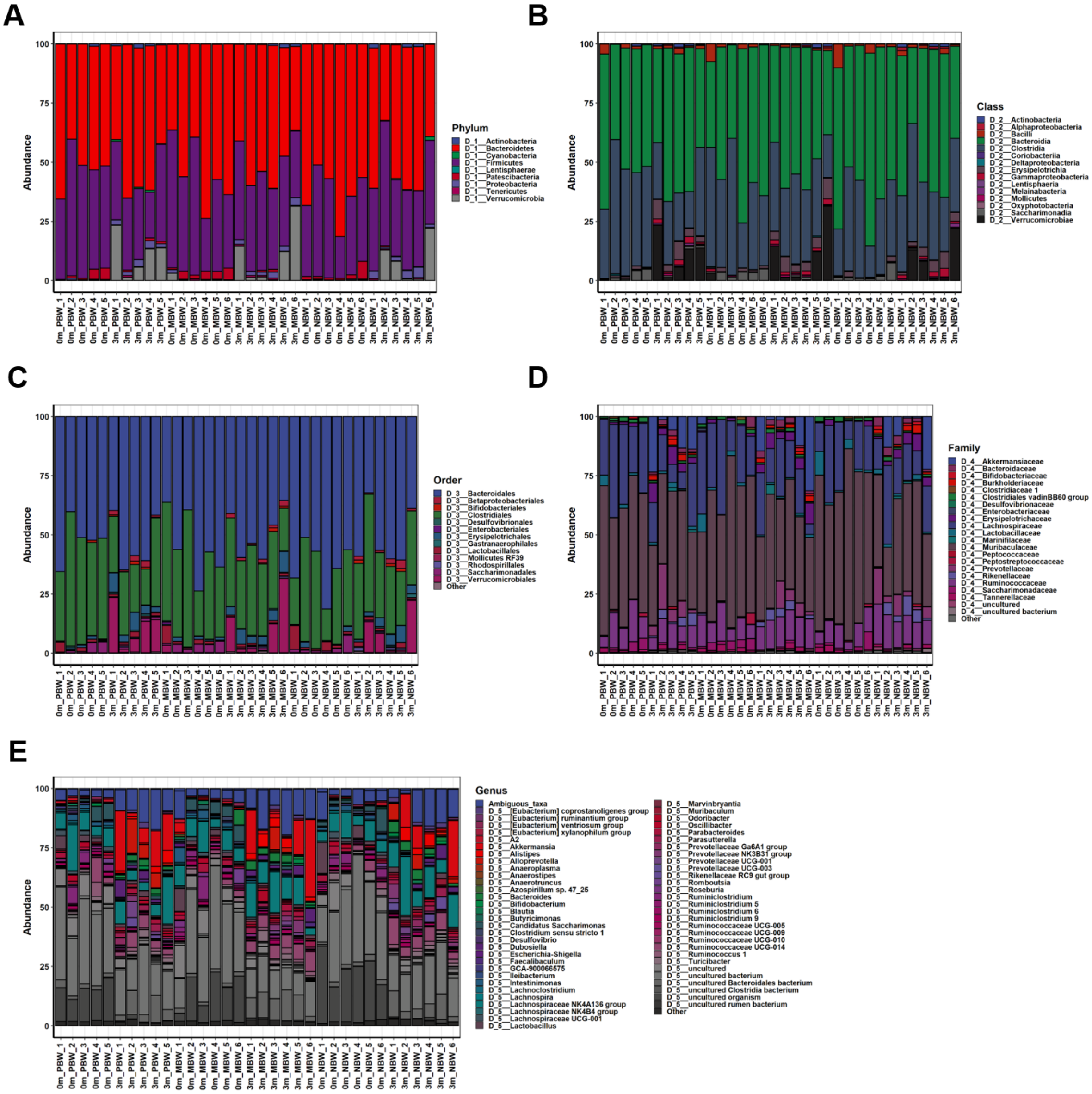

Figure S5. Changes in the composition of the gut microbiome of each mouse before and after the gut microbiome replacement. **(A)** Phylum level, **(B)** Class level, **(C)** Order level, **(D)** Family level, and **(E)** Genus level are shown.

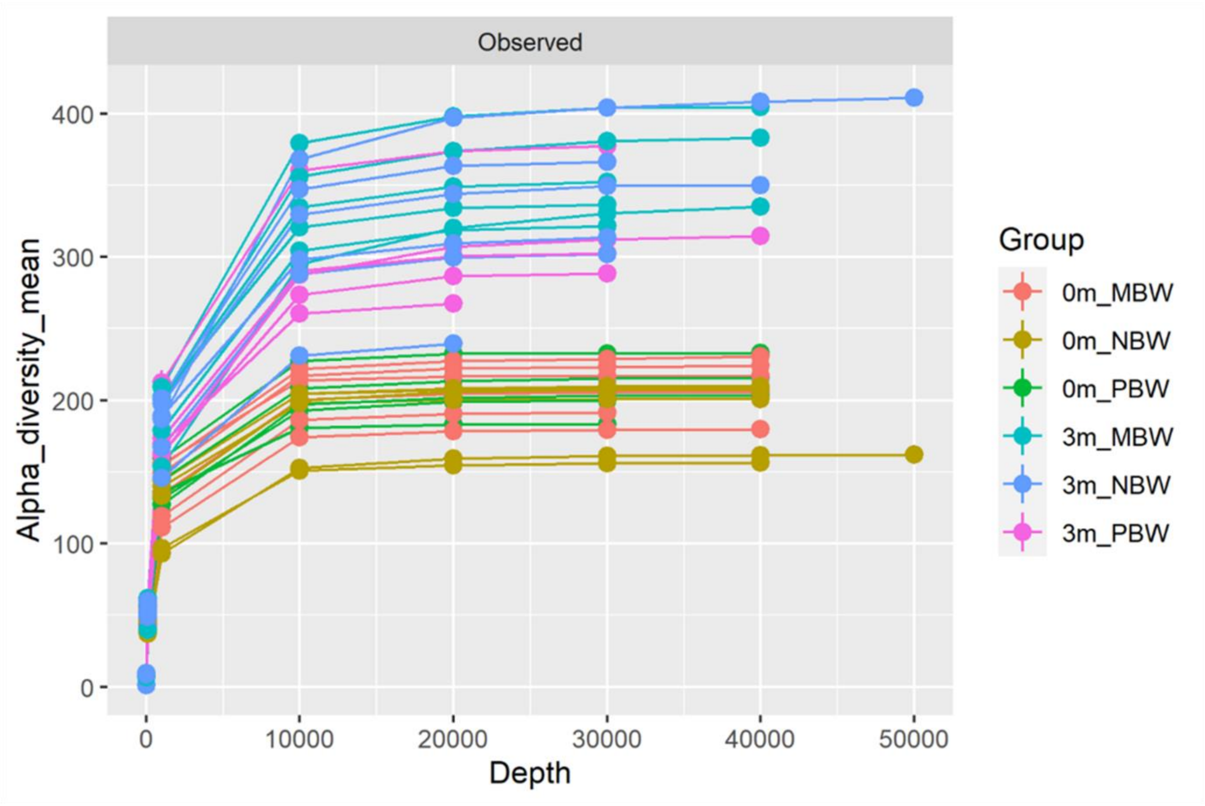

Figure S6. Rarefaction curves of each sample. Rarefaction curve constructed based on observed richness.

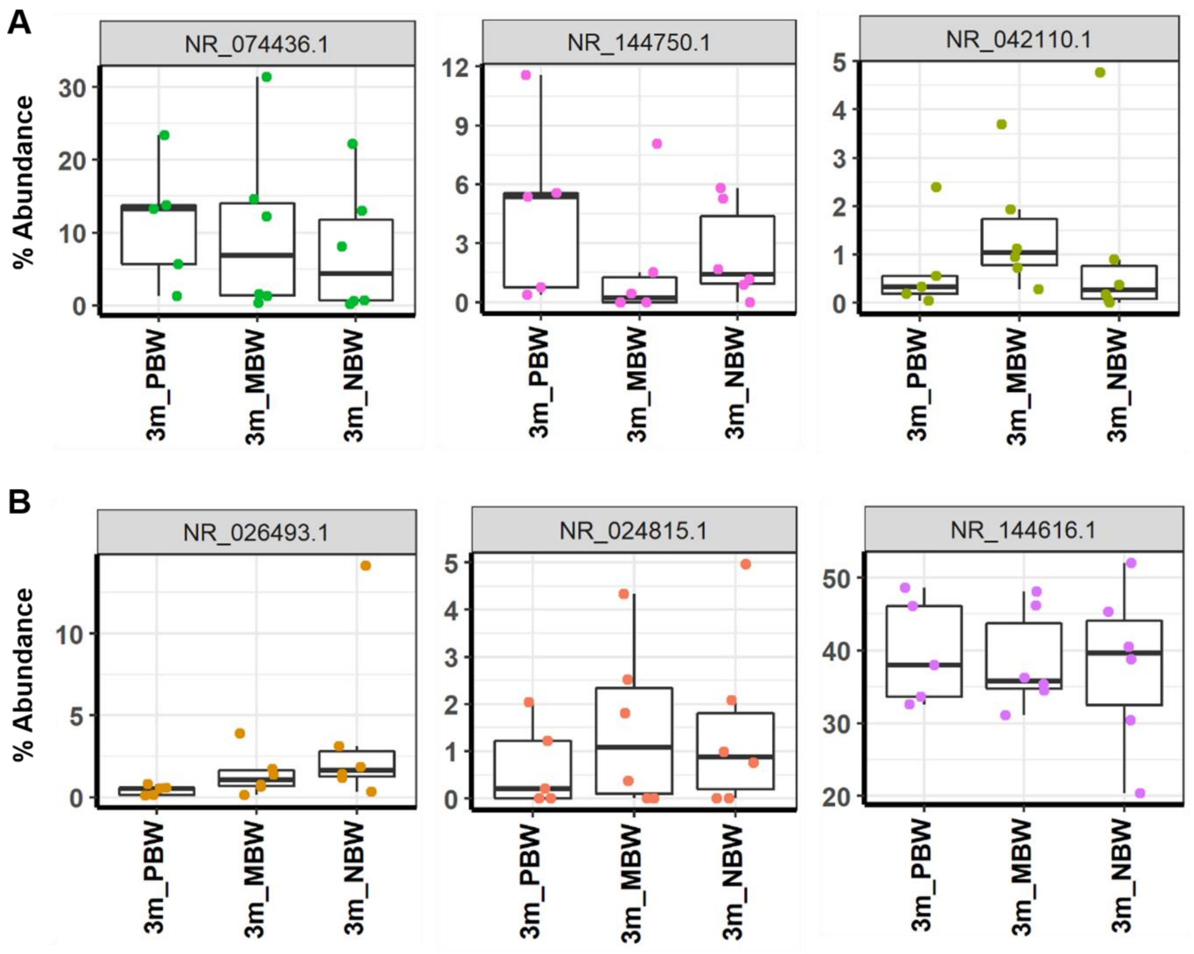

Figure S7. The relative abundance of the top 3 relatively abundant gut microbiota at the species level in PBW and NBW groups from 3 month after gut microbiome replacement. **(A)** Relative distribution by sample group of the species more abundant in the PBW group than in the NBW group. **(B)** Relative distribution by sample group of the species more abundant in the NBW group than in the PBW group.

Table S1. The valid reads of the 16S rRNA amplicon sequence.

Group	0m_PBW	0m_MBW	0m_NBW	3m_PBW	3m_MBW	3m_NBW
**Total reads**	42029 ± 3363	43137 ± 1116	44629 ± 1727	33441 ± 2994	40193 ± 2538	37144 ± 4007

All values are mean ±SEM.OTUs,operationaltaxonomic units.

Table S2. Comparison of taxonomy abundance at the phylum level.

	Phylum	0m_PBW (%)	0m_MBW (%)	0m_NBW (%)	3m_ PBW (%)	3m_ MBW (%)	3m_ NBW (%)	Total (%)
**1**	**D_1__Verrucomicrobia**	0	0.52	0	11.48	10.23	7.44	4.94
**2**	**D_1__Bacteroidetes**	52.12	54.39	63.02	53.22	49.30	51.29	53.89
**3**	**D_1__Firmicutes**	44.99	41.44	34.23	30.73	36.61	36.85	37.47
**4**	**D_1__Patescibacteria**	2.16	2.89	2.21	0.66	0.37	0.21	1.42
**5**	**D_1__Actinobacteria**	0.23	0.07	0.08	0.81	0.66	0.84	0.450
**6**	**D_1__Proteobacteria**	0.32	0.47	0.33	2.22	2.17	2.45	1.33
**7**	**D_1__Tenericutes**	0.18	0.22	0.14	0.37	0.54	0.40	0.308
**8**	**D_1__Cyanobacteria**	0	0.002	0	0.51	0.11	0.51	0.189
**9**	**D_1__Lentisphaerae**	0	0	0	0.001	0.01	0	0.001

Table S3. Comparison of taxonomy abundance at the class level.

	Class	0m_ PBW (%)	0m_ MBW (%)	0m_ NBW (%)	3m_ PBW (%)	3m_ MBW (%)	3m_ NBW (%)
**1**	**D_2__Verrucomicrobiae**	0	0.52	0	11.48	10.23	7.44
**2**	**D_2__Bacteroidia**	52.12	54.39	63.02	53.22	49.30	51.29
**3**	**D_2__Clostridia**	42.65	38.42	30.35	24.61	30.80	31.96
**4**	**D_2__Erysipelotrichia**	0.79	0.90	0.92	4.86	4.99	3.63
**5**	**D_2__Saccharimonadia**	2.16	2.89	2.21	0.66	0.37	0.21
**6**	**D_2__Bacilli**	1.55	2.12	2.96	1.26	0.82	1.26
**7**	**D_2__Actinobacteria**	0.18	0.007	0.00	0.66	0.56	0.67
**8**	**D_2__Coriobacteriia**	0.05	0.07	0.08	0.15	0.10	0.18
**9**	**D_2__Deltaproteobacteria**	0.22	0.15	0.17	0.18	0.27	0.15
**10**	**D_2__Gammaproteobacteria**	0.08	0.28	0.10	1.66	1.52	1.60
**11**	**D_2__Mollicutes**	0.18	0.22	0.14	0.37	0.54	0.40
**12**	**D_2__Alphaproteobacteria**	0.02	0.05	0.06	0.38	0.38	0.70
**13**	**D_2__Melainabacteria**	0	0	0	0.51	0.10	0.51
**14**	**D_2__Oxyphotobacteria**	0	0.002	0	0.001	0.004	0.004
**15**	**D_2__Lentisphaeria**	0	0	0	0.001	0.005	0

Table S4. Comparison of taxonomy abundance at the order level.

	Order	0m_ PBW (%)	0m_ MBW (%)	0m_ NBW (%)	3m_ PBW (%)	3m_ MBW (%)	3m_ NBW (%)
**1**	**D_3__Verrucomicrobiales**	0	0.52	0	11.48	10.23	7.44
**2**	**D_3__Bacteroidales**	52.12	54.38	63.02	53.21	49.28	51.25
**3**	**D_3__Clostridiales**	42.65	38.42	30.35	24.61	30.80	31.96
**4**	**D_3__Erysipelotrichales**	0.79	0.90	0.92	4.86	4.99	3.63
**5**	**D_3__Saccharimonadales**	2.16	2.89	2.21	0.66	0.37	0.21
**6**	**D_3__Lactobacillales**	1.55	2.12	2.96	1.26	0.82	1.26
**7**	**D_3__Bifidobacteriales**	0.18	0.01	0	0.66	0.56	0.67
**8**	**D_3__Desulfovibrionales**	0.22	0.15	0.17	0.18	0.27	0.15
**9**	**D_3__Betaproteobacteriales**	0	0.01	0	1.64	1.51	1.59
**10**	**D_3__Mollicutes RF39**	0.18	0.22	0.14	0.23	0.27	0.22
**11**	**D_3__Enterobacteriales**	0.04	0.22	0.05	0.007	0.009	0.008
**12**	**D_3__Rhodospirillales**	0	0	0	0.37	0.37	0.69
**13**	**D_3__Gastranaerophilales**	0	0	0	0.51	0.10	0.51
**14**	**Other < 0.5%**	0.11	0.18	0.19	0.32	0.43	0.41

Table S5. Comparison of taxonomy abundance at the family level.

	Family	0m_ PBW (%)	0m_ MBW (%)	0m_ NBW (%)	3m_ PBW (%)	3m_ MBW (%)	3m_ NBW (%)
**1**	**D_4__Akkermansiaceae**	0	0.52	0	11.48	10.23	7.45
**2**	**D_4__Muribaculaceae**	50.92	51.90	61.60	39.81	38.65	37.93
**3**	**D_4__Lachnospiraceae**	29.14	25.88	20.04	14.75	17.32	17.69
**4**	**D_4__Erysipelotrichaceae**	0.79	0.90	0.92	4.86	4.99	3.63
**5**	**D_4__Saccharimonadaceae**	2.16	2.89	2.21	0.66	0.37	0.21
**6**	**D_4__Tannerellaceae**	0.95	1.40	1.21	0.80	0.97	0.77
**7**	**D_4__Lactobacillaceae**	1.54	2.08	2.95	1.25	0.79	1.26
**8**	**D_4__Ruminococcaceae**	10.96	9.69	7.92	8.34	12.11	12.62
**9**	**D_4__Prevotellaceae**	0	0	0	7.35	3.95	5.81
**10**	**D_4__Bacteroidaceae**	0	0.78	0	1.78	2.13	2.61
**11**	**D_4__Clostridiales vadinBB60 group**	1.42	1.12	1.36	0.73	0.92	0.82
**12**	**D_4__Rikenellaceae**	0.28	0.32	0.22	2.87	2.66	3.65
**13**	**D_4__Bifidobacteriaceae**	0.19	0.01	0	0.66	0.56	0.67
**14**	**D_4__Desulfovibrionaceae**	0.22	0.15	0.17	0.18	0.27	0.15
**15**	**D_4__Burkholderiaceae**	0	0.007	0.00	1.65	1.51	1.60
**16**	**D_4__Peptococcaceae**	0.22	0.18	0.15	0.47	0.23	0.38
**17**	**D_4__Clostridiaceae 1**	0.29	0.44	0.31	0.13	0.07	0.22
**18**	**D_4__Marinifilaceae**	0	0	0	0.64	0.96	0.52
**19**	**D_4__Peptostreptococcaceae**	0.48	0.92	0.39	0.04	0	0.06
**20**	**D_4__uncultured bacterium**	0.12	0.18	0.07	0.07	0.14	0.06
**21**	**D_4__Enterobacteriaceae**	0.04	0.22	0.05	0.007	0.009	0.008
**22**	**D_4__uncultured**	0	0	0	0.37	0.37	0.69
**23**	**D_4__uncultured bacterium**	0	0	0	0.46	0.08	0.47
**24**	**Other < 0.5%**	0.29	0.41	0.45	0.63	0.70	0.72

Table S6. Comparison of taxonomy abundance at the genus level.

	Genus	0m_ PBW (%)	0m_ MBW (%)	0m_ NBW (%)	3m_ PBW (%)	3m_ MBW (%)	3m_ NBW (%)
1	D_5__Akkermansia	0	0.66	0	12.66	11.50	8.15
2	Ambiguous_taxa	3.21	5.15	5.41	11.53	12.34	10.08
3	D_5__uncultured organism	13.77	13.90	22.18	0.48	0.34	0.52
4	D_5__uncultured bacterium	31.40	29.50	29.88	18.08	12.05	17.89
5	D_5__Turicibacter	0.93	1.09	1.18	1.71	1.82	1.27
6	D_5__uncultured Bacteroidales bacterium	1.97	1.65	1.95	6.43	9.07	6.19
7	D_5__Lachnospiraceae NK4A136 group	9.13	7.79	6.62	8.42	10.77	12.35
8	D_5__[Eubacterium] coprostanoligenes group	0.04	0.04	0.04	0.92	0.80	1.10
9	D_5__Candidatus Saccharimonas	3.07	3.93	2.92	0.76	0.44	0.24
10	D_5__Parabacteroides	1.28	1.86	1.60	0.94	1.16	0.85
11	D_5__Lactobacillus	2.08	2.74	3.93	1.39	0.91	1.42
12	D_5__Ruminiclostridium	3.30	3.06	2.37	0.44	0.82	0.47
13	D_5__Alloprevotella	0	0	0	5.52	1.97	2.87
14	D_5__Bacteroides	0	1.09	0	2.01	2.52	2.97
15	D_5__uncultured bacterium	1.06	0.81	0.70	0.35	0.57	0.42
16	D_5__Alistipes	0.37	0.43	0.29	2.18	2.99	2.46
17	D_5__Faecalibaculum	0	0.02	0.005	0.52	0.56	0.40
18	D_5__Prevotellaceae UCG-001	0	0	0	1.95	0.75	1.01
19	D_5__Muribaculum	0	0.08	0	1.14	0.87	1.10
20	D_5__Bifidobacterium	0.26	0.009	0	0.73	0.64	0.76
21	D_5__Intestinimonas	1.53	2.33	1.46	0.18	0.24	0.21
22	D_5__Dubosiella	0	0	0	2.80	2.92	2.09
23	D_5__Desulfovibrio	0.28	0.19	0.22	0.02	0.07	0.01
24	D_5__Ruminococcaceae UCG-014	2.73	1.35	1.78	3.82	6.00	6.08
25	D_5__uncultured	6.95	6.47	5.34	1.58	1.76	1.26
26	D_5__[Eubacterium] ventriosum group	0.26	0.23	0.26	0	0	0.006
27	D_5__Prevotellaceae NK3B31 group	0	0	0	0.52	1.72	1.62
28	D_5__[Eubacterium] ruminantium group	0	0	0	0.16	0	0.36
29	D_5__uncultured	0.57	0.53	0.41	0.32	0.86	1.07
30	Ambiguous_taxa	0.44	0.42	0.64	0.32	0.29	0.35
31	D_5__Blautia	0.34	0.69	0.40	0.20	0.36	0.26
32	D_5__Ruminiclostridium 9	1.36	1.29	1.10	0.55	1.13	1.06
33	D_5__Rikenellaceae RC9 gut group	0	0	0	1.12	0.13	1.44
34	D_5__uncultured	0	0	0	0.12	0.14	0.04
35	D_5__[Eubacterium] xylanophilum group	0.46	0.52	0.41	0.05	0.41	0.21
36	D_5__Lachnospiraceae UCG-001	1.65	1.07	0.97	0.19	0.23	0.14
37	D_5__Oscillibacter	1.41	1.07	0.95	0.48	0.61	0.67
38	D_5__uncultured Clostridia bacterium	0.11	0.06	0.21	0.006	0.01	0.009
39	D_5__Marvinbryantia	0.33	0.29	0.24	0.02	0.14	0.02
40	D_5__Parasutterella	0	0	0	1.83	1.74	1.77
41	D_5__Ruminococcaceae UCG-009	0.45	0.27	0.28	0.14	0.17	0.16
42	D_5__uncultured	0.27	0.22	0.18	0.50	0.25	0.41
43	D_5__Ruminococcus 1	1.20	0.06	0	1.02	1.23	1.12
44	D_5__Prevotellaceae Ga6A1 group	0	0	0	0.61	0.16	0.67
45	D_5__Ruminiclostridium 6	0.28	0.37	0.43	0.31	0.35	0.33
46	D_5__Clostridium sensu stricto 1	0.31	0.59	0.42	0.05	0.02	0.17
47	D_5__Roseburia	0.53	0.99	0.53	0.26	0.17	0.21
48	D_5__Lachnoclostridium	0.82	0.74	0.41	0.09	0.40	0.20
49	D_5__GCA-900066575	0.66	0.48	0.44	0.15	0.17	0.13
50	D_5__Odoribacter	0	0	0	0.68	1.06	0.45
51	D_5__Ileibacterium	0	0	0	0.12	0.23	0.08
52	D_5__A2	0.97	0.63	0.46	0.02	0.20	0.06
53	D_5__Ruminiclostridium 5	0.47	0.85	0.43	0.09	0.18	0.20

**54**	**D_5__Anaeroplasma**	0	0	0	0.16	0.31	0.20
**55**	**D_5__Lachnospira**	0.39	0.36	0.27	0.003	0.004	0.004
**56**	**D_5__Romboutsia**	0.68	1.27	0.52	0.04	0	0.08
**57**	**D_5__Anaerostipes**	0	0	0	0.08	0.14	0.012
**58**	**D_5__uncultured**	0.05	0.08	0.001	0.15	0.14	0.10
**59**	**D_5__Anaerotruncus**	0.16	0.34	0.18	0.06	0.07	0.04
**60**	**D_5__uncultured bacterium**	0.16	0.25	0.09	0.08	0.17	0.07
**61**	**D_5__Azospirillum sp. 47_25**	0	0	0	0.20	0.23	0.18
**62**	**D_5__Escherichia-Shigella**	0.04	0.26	0.04	0.003	0.01	0.009
**63**	**D_5__Ruminococcaceae UCG-010**	0.10	0.03	0.04	0.27	0.21	0.28
**64**	**D_5__Prevotellaceae UCG-003**	0	0	0	0.009	0.06	0.53
**65**	**D_5__Butyricimonas**	0	0	0	0.02	0.05	0.14
**66**	**D_5__uncultured bacterium**	0	0	0	0.51	0.09	0.51
**67**	**D_5__uncultured rumen bacterium**	0	0	0	0.12	0.05	0.14
**68**	**D_5__uncultured bacterium**	0	0	0	0.06	0.05	0.12
**69**	**D_5__Lachnospiraceae NK4B4 group**	0.20	0.11	0.14	0	0	0
**70**	**D_5__Ruminococcaceae UCG-005**	0.15	0.05	0.03	0.03	0.04	0.03
**71**	**Other < 0.5%**	1.83	1.73	1.68	1.69	2.09	2.19

Table S7. Comparison of taxonomy abundance at the species level.

	Species	0m_ PBW (%)	0m_ MBW (%)	0m_ NBW (%)	3m_ PBW (%)	3m_ MBW (%)	3m_ NBW (%)
**1**	**D_6__uncultured bacterium**	0	0.90	0	14.55	13.67	7.41
**2**	**Ambiguous_taxa**	3.83	6.44	6.27	13.43	14.75	12.42
**3**	**D_6__uncultured organism**	16.44	16.27	25.67	0.58	0.41	0.62
**4**	**D_6__uncultured bacterium**	37.55	34.49	34.38	21.29	14.46	22.34
**5**	**D_6__Turicibacter sp. LA61**	1.16	1.26	1.37	2.03	2.16	1.58
**6**	**D_6__uncultured Bacteroidales bacterium**	2.34	2.01	2.25	7.49	10.94	7.70
**7**	**D_6__Trichinella pseudospiralis**	0	0	0	0.26	1.58	4.84
**8**	**D_6__Lachnospiraceae bacterium 10-1**	0	0	0	2.13	0.45	1.65
**9**	**D_6__uncultured bacterium**	3.77	4.65	3.34	0.88	0.54	0.29
**10**	**D_6__uncultured Parabacteroides sp.**	1.48	2.25	1.88	0	0.02	0.01
**11**	**D_6__Lactobacillus murinus**	2.09	1.06	2.33	0.48	0.22	0.53
**12**	**D_6__uncultured rumen bacterium**	0.37	0	0	0.60	1.01	1.43
**13**	**D_6__uncultured bacterium**	3.97	3.65	2.72	0.49	0.97	0.59
**14**	**D_6__gut metagenome**	0	0	0	6.57	2.42	3.64
**15**	**D_6__uncultured bacterium**	1.28	0.98	0.81	0.40	0.69	0.54
**16**	**D_6__uncultured bacterium**	0	0.01	0.007	1.88	2.96	2.39
**17**	**D_6__uncultured bacterium**	3.97	4.77	2.73	2.35	3.27	2.37
**18**	**D_6__uncultured bacterium**	0	0.02	0.005	0.60	0.66	0.48
**19**	**D_6__uncultured bacterium**	0	0.10	0	1.35	1.05	1.37
**20**	**D_6__uncultured bacterium**	1.84	2.88	1.67	0.09	0.13	0.09
**21**	**D_6__uncultured bacterium**	0	0	0	3.16	3.43	1.49
**22**	**D_6__uncultured bacterium**	0.35	0.24	0.26	0.03	0.08	0.01
**23**	**D_6__Clostridiales bacterium CIEAF 020**	0	0	0	0.33	0.86	1.28
**24**	**D_6__Bacteroides caecimuris**	0	0	0	0.61	0.48	0.29
**25**	**D_6__Bacteroides acidifaciens**	0	0	0	0.38	0.25	0.09
**26**	**D_6__uncultured bacterium**	0.61	0.64	0.69	0.36	0.43	0.57
**27**	**D_6__uncultured bacterium**	0	0	0	0.63	2.07	2.11
**28**	**D_6__uncultured bacterium**	3.94	3.55	2.53	0.96	1.29	1.08
**29**	**D_6__uncultured bacterium**	0	0	0	0.20	0	0.45
**30**	**D_6__Clostridium sp. Culture-1**	0.23	0.18	0.18	0.15	0.61	1.11
**31**	**Ambiguous_taxa**	0.53	0.53	0.75	0.37	0.35	0.44
**32**	**D_6__unidentified**	0.43	0.25	0.27	0.35	0.44	0.27
**33**	**D_6__uncultured Bacteroidales bacterium**	0	0	0.00	0.96	1.13	0.76
**34**	**D_6__uncultured Clostridiales bacterium**	0.81	0.49	0.36	1.18	1.80	0.14
**35**	**Ambiguous_taxa**	0	0	0	1.19	0.16	1.69
**36**	**Ambiguous_taxa**	0	0	0	0.14	0.17	0.05
**37**	**D_6__uncultured bacterium**	0.56	0.61	0.47	0.06	0.50	0.26
**38**	**D_6__uncultured bacterium**	1.94	1.25	1.13	0.23	0.25	0.18
**39**	**D_6__uncultured organism**	0	0	0	0.41	0.11	0.34
**40**	**D_6__unidentified**	0.83	0.44	0.27	0.18	0.16	0.15
**41**	**D_6__Parabacteroides distasonis**	0	0	0	0.05	0.14	0.18
**42**	**D_6__Lachnospiraceae bacterium COE1**	0.34	0.50	0.38	0.08	0.47	0.32
**43**	**D_6__uncultured Clostridia bacterium**	0.12	0.06	0.24	0.008	0.01	0.01
**44**	**D_6__uncultured bacterium**	0.32	0.28	0.21	0.00	0.15	0.00
**45**	**D_6__Bacteroides dorei**	0	0	0	0.07	0.06	0.27
**46**	**D_6__uncultured bacterium**	0	0	0	1.20	0.29	0.63
**47**	**Ambiguous_taxa**	0.54	0.32	0.33	0.16	0.20	0.19
**48**	**D_6__uncultured bacterium**	0.32	0.26	0.21	0.18	0.20	0.17
**49**	**D_6__Firmicutes bacterium M10-2**	0	0	0	0	0	1.03
**50**	**Ambiguous_taxa**	0	0	0	0.69	0.18	0.85
**51**	**D_6__uncultured bacterium**	0.12	0.23	0.43	0.39	1.11	1.11
**52**	**D_6__uncultured bacterium**	0	0	0	0.29	0.30	0.42

**53**	**D_6__uncultured bacterium**	0.69	0.55	0.50	0.37	0.78	0.85
**54**	**D_6__uncultured bacterium**	0.38	0.70	0.48	0.06	0.03	0.22
**55**	**D_6__uncultured bacterium**	0.39	0.86	0.33	0.09	0.09	0.12
**56**	**D_6__uncultured bacterium**	0.56	0.43	0.18	0.05	0.32	0.19
**57**	**D_6__uncultured bacterium**	0.56	0.43	0.35	0.08	0.17	0.15
**58**	**D_6__uncultured bacterium**	0	0	0	0.57	0.95	0.21
**59**	**D_6__Lactobacillus reuteri**	0.04	0.16	0.20	0.18	0.07	0.13
**60**	**Ambiguous_taxa**	0	0	0	0.14	0.28	0.09
**61**	**D_6__Lachnospiraceae bacterium A2**	0.75	0.51	0.37	0	0.01	0.006
**62**	**Ambiguous_taxa**	0.47	0.71	0.38	0.03	0.09	0.07
**63**	**D_6__uncultured bacterium**	0	0	0	0.19	0.38	0.24
**64**	**D_6__uncultured bacterium**	0	0	0	0.34	0.09	0.14
**65**	**D_6__Burkholderiales bacterium YL45**	0	0	0	0.69	1.27	0.87
**66**	**D_6__mouse gut metagenome**	0.16	0.28	0.17	0	0	0
**67**	**Ambiguous_taxa**	0	0	0	0.03	0.13	0.01
**68**	**D_6__uncultured bacterium**	0.07	0.11	0.002	0.17	0.17	0.13
**69**	**D_6__uncultured bacterium**	0.20	0.43	0.20	0.08	0.09	0.05
**70**	**Ambiguous_taxa**	0	0.00	0	0.24	0.27	0.33
**71**	**D_6__Clostridium sp. Culture-27**	0.46	0.24	0.38	0.02	0.05	0.01
**72**	**D_6__uncultured bacterium**	0.19	0.29	0.10	0.10	0.20	0.09
**73**	**D_6__Azospirillum sp. 47_25**	0	0	0	0.24	0.27	0.22
**74**	**D_6__gut metagenome**	0	0	0	0.35	0.24	0.38
**75**	**D_6__uncultured bacterium**	0	0	0	0.01	0.07	0.70
**76**	**D_6__uncultured bacterium**	0.42	0.28	0.16	0.03	0.05	0.07
**77**	**D_6__uncultured bacterium**	0	0	0	0	0	0.28
**78**	**D_6__uncultured rumen bacterium**	0.03	0	0.09	0.11	0.08	0.06
**79**	**D_6__uncultured bacterium**	0	0	0	0.59	0.11	0.67
**80**	**D_6__uncultured rumen bacterium**	0	0	0	0.14	0.06	0.16
**81**	**Ambiguous_taxa**	0	0	0	0	0	0.13
**82**	**D_6__uncultured organism**	0	0	0	0.13	0.04	0.02
**83**	**D_6__uncultured bacterium**	0	0	0	0.25	0.18	0.12
**84**	**D_6__Bacteroides massiliensis B84634**	0	0	0	0.03	0.30	0.20
**85**	**D_6__uncultured bacterium**	0	0	0	0.07	0.06	0.14
**86**	**D_6__uncultured bacterium**	0.23	0.14	0.16	0	0	0
**87**	**Ambiguous_taxa**	0	0	0	0	0	0.11
**88**	**D_6__uncultured bacterium**	0.02	0.15	0.02	0.02	0.00	0.009
**89**	**D_6__uncultured bacterium**	0	0	0	0.13	0	0
**90**	**D_6__uncultured Clostridiales bacterium**	0	0	0	0.07	0.02	0.15
**91**	**D_6__Clostridium sp. Clone-44**	0.02	0.12	0.11	0	0	0
**92**	**Ambiguous_taxa**	0	0	0	0	0.18	0
**93**	**D_6__uncultured bacterium**	0	0	0	0	0	0.09
**94**	**Other < 0.5%**	2.27	2.02	1.69	1.98	2.89	2.31

Table S8. The α-diversity indexes for each sample and group.

Sample	Observed	ACE	Shannon	Simpson	InvSimpson	Fisher
**0m_ PBW _1**	204	204.15	3.99	0.961	25.94	27.52
**0m_ PBW _2**	234	234.00	4.52	0.980	49.80	31.97
**0m_ PBW _3**	217	217.43	4.16	0.967	29.92	29.26
**0m_ PBW _4**	201	201.00	4.19	0.973	36.42	28.32
**0m_ PBW _5**	184	184.25	4.18	0.969	32.40	25.63
**0m_MBW_1**	225	225.48	4.30	0.973	37.32	31.04
**0m_ MBW _2**	206	206.15	4.10	0.965	28.67	28.21
**0m_ MBW _3**	217	217.00	4.44	0.978	44.45	29.98
**0m_ MBW _4**	181	181.21	3.83	0.957	23.27	23.87
**0m_ MBW _5**	232	232.76	4.36	0.974	38.29	31.95
**0m_ MBW _6**	194	194.64	4.03	0.966	29.34	26.57
**0m_NBW_1**	158	158.32	3.57	0.935	15.30	20.55
**0m_ NBW _2**	212	212.47	4.29	0.972	36.12	29.26
**0m_ NBW _3**	201	201.00	4.03	0.960	25.02	27.58
**0m_ NBW _4**	162	162.00	3.64	0.952	20.98	20.68
**0m_ NBW _5**	208	208.00	4.02	0.958	23.63	28.25
**0m_ NBW _6**	210	210.00	4.26	0.973	37.50	28.51
**3m_ PBW _1**	270	270.76	4.12	0.935	15.28	43.19
**3m_ PBW _2**	305	305.46	4.39	0.969	32.70	46.08
**3m_ PBW _3**	291	291.31	4.34	0.969	32.71	43.50
**3m_ PBW _4**	318	319.11	3.97	0.946	18.43	47.03
**3m_ PBW _5**	383	384.08	4.66	0.969	31.93	60.19
**3m_ MBW _1**	356	356.61	4.62	0.968	31.15	54.17
**3m_ MBW _2**	410	411.15	4.66	0.972	35.20	62.43
**3m_ MBW _3**	339	339.73	4.76	0.981	52.98	52.19
**3m_ MBW _4**	386	386.31	4.61	0.969	32.24	59.04
**3m_ MBW _5**	326	326.56	4.43	0.967	29.89	50.19
**3m_ MBW _6**	343	344.93	3.58	0.882	8.49	49.63
**3m_ NBW _1**	318	320.19	4.71	0.983	58.02	48.07
**3m_ NBW _2**	307	309.10	4.23	0.955	22.34	46.82
**3m_ NBW _3**	371	371.48	4.68	0.976	41.95	57.05
**3m_ NBW _4**	352	352.16	4.63	0.973	36.62	53.01
**3m_ NBW _5**	246	249.46	4.28	0.970	33.60	38.53
**3m_ NBW _6**	418	420.38	4.27	0.957	23.18	61.94

Group	Observed	ACE	Shannon	Simpson	InvSimpson	Fisher
**0m_PBW**	208	208.17	4.21	0.970	34.89	28.54
**0m_MBW**	209.17	209.54	4.18	0.969	33.56	28.60
**0m_NBW**	191.83	191.97	3.97	0.958	26.43	25.81
**3m_ PBW**	313.40	314.14	4.30	0.958	26.21	48.00
**3m_ MBW**	360	360.88	4.44	0.956	31.66	54.61
**3m_ NBW**	335.33	337.13	4.47	0.969	35.95	50.90

Table S9. The co-occurrence network indices.

Network indexes indices	0m_ PBW	0m_ MBW	0m_ NBW	3m_ PBW	3m_ MBW	3m_ NBW
Node	120	120	113	150	157	158
Edge	133	123	129	138	153	149
Network Density	0.019	0.017	0.020	0.012	0.012	0.012
Assortativity	-0.013	-0.013	-0.015	-0.009	-0.009	-0.008
Module	34	37	33	45	30	47
Modularity	0.894	0.895	0.817	0.929	0.951	0.957

Table S10. The taxonomic classification of the intestinal microbes most different between PBW and NBW groups.

**Most abundant in PBW**			
**Rank**	**1**	**2**	**3**
**OUT**	OTU0001	OTU0039	OTU0083
**Kingdom**	D_0__Bacteria	D_0__Bacteria	D_0__Bacteria
**Phylum**	D_1__Verrucomicrobia	D_1__Bacteroidetes	D_1__Firmicutes
**Class**	D_2__Verrucomicrobiae	D_2__Bacteroidia	D_2__Erysipelotrichia
**Order**	D_3__Verrucomicrobiales	D_3__Bacteroidales	D_3__Erysipelotrichales
**Family**	D_4__Akkermansiaceae	D_4__Prevotellaceae	D_4__Erysipelotrichaceae
**Genus**	D_5__Akkermansia	D_5__Alloprevotella	D_5__Dubosiella
**Species**	**D_6__uncultured bacterium**	**D_6__uncultured bacterium**	**D_6__uncultured bacterium**
**sseqid**	NR_074436.1	NR_144750.1	NR_042110.1

**Most abundant in NBW**			
**Rank**	**1**	**2**	**3**
**OUT**	OTU0024	OTU0108	OTU0004
**Kingdom**	D_0__Bacteria	D_0__Bacteria	D_0__Bacteria
**Phylum**	D_1__Firmicutes	D_1__Bacteroidetes	D_1__Bacteroidetes
**Class**	D_2__Clostridia	D_2__Bacteroidia	D_2__Bacteroidia
**Order**	D_3__Clostridiales	D_3__Bacteroidales	D_3__Bacteroidales
**Family**	D_4__Lachnospiraceae	D_4__Prevotellaceae	D_4__Muribaculaceae
**Genus**	D_5__Lachnospiraceae NK4A136 group	D_5__Prevotellaceae NK3B31 group	D_5__uncultured bacterium
**Species**	**D_6__Trichinella pseudospiralis**	**D_6__uncultured bacterium**	**D_6__uncultured bacterium**
**sseqid**	NR_026493.1	NR_024815.1	NR_144616.1
